# The Annual Conventions

**Published:** 1895-07

**Authors:** 


### THE ANNUAL CONVENTIONS.

The period for the convening of the members of the various conventions is near at hand. We presume all who have, heretofore, made it a part of their yearly duties to be present at one or more of these will not neglect it now. There is a large indifferent class who reluctantly go anywhere, and especially to a dental convention. These need to be infused with a higher degree of professional spirit, and this can best be secured by commingling with their fellows in meetings of this character.

The American Dental Association meets this year at Asbury Park. This place, as a sea side resort, has no superior on the New Jersey coast, and the occasion should be made an enjoyable one aside from that derived from professional association. Every effort is being put forth to make it a memorable meeting. The promise of a large attendance seems to be assured.

The several bodies which annually gather at the same place, as the American Dental Association, the National Association of Dental Faculties, and the National Association of Dental Examiners, will be fully attended. These organizations are growing yearly in importance and power, and will require wise management that this be not exceeded.

The annual State meetings occurring at this season of the year will be held at periods not to interfere with the National organization.

The meeting of the Southern Dental Association will take place October 1.

The American Dental Society of Europe will meet at Boulogne-sur-Mer, France, August 5, 6, and 7, 1895. Those of the "professional brethren who may be spending their holidays in Europe are cordially invited to make their plans to be present."

